# Risk of HBV reactivation in patients with immune checkpoint inhibitor-treated unresectable hepatocellular carcinoma

**DOI:** 10.1136/jitc-2020-001072

**Published:** 2020-08-30

**Authors:** Pei-Chang Lee, Yee Chao, Ming-Huang Chen, Keng-Hsin Lan, I-Cheng Lee, Ming-Chih Hou, Yi-Hsiang Huang

**Affiliations:** 1 Division of Gastroenterology and Hepatology, Taipei Veterans General Hospital, Taipei, Taiwan; 2 Faculty of Medicine, National Yang-Ming University School of Medicine, Taipei, Taiwan; 3 Institute of Pharmacology, National Yang-Ming University School of Medicine, Taipei, Taiwan; 4 Department of Oncology, Taipei Veterans General Hospital, Taipei, Taiwan; 5 Institute of Clinical Medicine, National Yang-Ming University School of Medicine, Taipei, Taiwan

**Keywords:** liver neoplasms, immunotherapy

## Abstract

**Background:**

Immunotherapy with immune checkpoint inhibitor (ICI) is a promising treatment for unresectable hepatocellular carcinoma (HCC). However, whether ICIs would have the risk of hepatitis B virus (HBV) reactivation and the necessary of nucleos(t)ide analogs (NUCs) prophylaxis are still unclear. We aimed to investigate the role of NUCs prophylaxis in HBV-infected patients who underwent ICIs treatment.

**Methods:**

The study was a retrospective prospective design to review and follow-up consecutive 62 patients with chronic hepatitis B or resolved HBV infection who had received ICIs treatment for the unresectable HCC. Of them, 60 patients with documented baseline serum HBV DNA value were classified into three categories according to the baseline HBV viral load and the status of antiviral therapy before ICI treatment. The clinical status, including tumor response, viral kinetics and liver function, was recorded and investigated.

**Results:**

No HBV reactivation occurred in the 35 patients with HBV DNA ≤100 IU/mL on NUCs therapy. Of the 19 patients with HBV DNA >100 IU/mL who started NUCs simultaneously with ICI treatment, none encountered HBV reactivation during the immunotherapy. Of the six HBV patients without NUCs treatment, three had a greater than 1 log decrease in HBV viral load, and one maintained his serum HBV DNA in undetectable status during ICI treatment. Eventually, one out of six experienced HBV reactivation after 9 weeks of ICI treatment.

**Conclusion:**

No patients on antiviral therapy developed HBV reactivation, and one out of six not receiving antiviral therapy had HBV reactivation. HBV viral load higher than 100 IU/mL is safe and not a contraindication for ICI treatment for HCC, if NUCs can be coadministrated.

## Introduction

Hepatocellular carcinoma (HCC) is the ﬁfth most common cancer and the second-leading cause of cancer-related death worldwide that constitutes 854 000 new cases and 810 000 deaths per year.^1^ Hepatitis B virus (HBV) infection is common across the world, and globally approximate 54% of HCCs are attributed to chronic HBV infection.^2^ In Asian-Pacific region, chronic hepatitis B is endemic and plays much more important role in the development of HCC and its complications.^3 4^ Despite the improvement in surveillance and treatment of viral hepatitis, many patients still present with or progress to unresectable or advanced disease and require systemic therapy.^2^ Immunotherapies with immune checkpoint inhibitors (ICIs), such as nivolumab and pembrolizumab, the antiprogrammed cell death-1 (PD-1) antibodies, are recently emerged, effective immunotherapeutic agents for HCC.^5–7^

HBV reactivation is defined as the abrupt reappearance or rise of HBV DNA in the serum of a patient with resolved or inactive chronic HBV infection.^8^ This event can be triggered by the administration of either anticancer agents, immunosuppressive or biological therapies.^8–11^ CD8 T cell exhaustion due to PD-1 upregulation is characterized in chronic viral infection, including chronic hepatitis B (CHB).^12 13^ Previous studies suggested that blockade of the axis of PD-1 and its ligand (PD-L1) could restore anti-HBV T cell responses, which could enhance the control of HBV.^14–16^ In CheckMate-040 trial, 3 of 51 HBV-HCC patients (6%) presented a 1 log decline of HBV surface antigen (HBsAg) level during nivolumab therapy.^5^ However, two case reports described HBV reactivation in patients received ICI treatment for lung cancer.^17 18^ Currently, most of the clinical trials, including CheckMate-040 and Keynote-224, request CHB patients should be on nucleos(t)ide analogs (NUCs) treatment and had a HBV viral load <100 IU/mL before receiving their first dose of ICI treatment.^5 6^ So far, whether immunotherapy with ICIs would have the risk of HBV reactivation and the necessary of NUCs prophylaxis are still unclear.^2 11 19^ In this study, we aimed to investigate the risk of HBV reactivation and the role of NUCs treatment in HCC patients with CHB or resolved HBV infection undergoing ICIs treatment.

## Materials and methods

### Patients

The study was a retrospective prospective design to review and follow-up consecutive 62 patients with CHB or resolved HBV infection who had received nivolumab or pembrolizumab treatment for the unresectable HCC in Taipei Veterans General Hospital from May 2017 to September 2019. Of them, 60 patients with documented baseline serum HBV DNA level and evaluable image studies following the immunotherapy were finally enrolled in this study. Thirty-three patients who underwent immunotherapy before the end of June 2019 were retrospective reviewed of medical records. Since July 2019, the rest of the patients were recruited in an immunotherapy biomarker study and had prospectively observational monitoring.

The diagnosis of HCC was according to the AASLD treatment guidelines.^20^ ICI treatment was administered for HCC patients with intermediate stage after locoregional treatment failure by transarterial chemoembolization (TACE), or advanced stage with intolerable adverse events or treatment failure to sorafenib, or deteriorated liver reserves beyond Child-Pugh class A so that target therapy was not approved based on the reimbursement criteria of National Health Insurance (NHI) in Taiwan.^21 22^ All the enrolled patients did not receive other locoregional therapies, including TACE, during the ICI treatment. According to their baseline HBV viral load and the status of antiviral therapy before ICI treatment, the enrolled patients were classified into three categories, including (1) patients with HBV DNA ≤100 IU/mL on NUCs therapy (fulfilled CheckMate-040 and Keynote-224 HBV criteria),^5 6^ (2) patients with HBV DNA >100 IU/mL on NUCs therapy and (3) patients with HBV but without receiving NUCs therapy throughout the ICI treatment because of not fulfilling the NUCs reimbursement criteria for CHB in NHI, Taiwan.

### Treatment and outcome assessment

ICIs were prescribed according to the recommended dosage and safety information (2–3 mg/kg, every 2 weeks for nivolumab and every 3 weeks for pembrolizumab).^5 6^ National Cancer Institute Common Terminology Criteria for Adverse Events (CTCAE; V.5.0) was applied for the assessment of therapeutic safety. Clinical evaluation with Child-Pugh class, albumin-bilirubin (ALBI) grade, hemogram, serum chemistry and alpha-fetoprotein (AFP) level were performed every 2–3 weeks during the treatment. Clinical tumor response was assessed by RECIST V.1.1 based on contrast-enhanced abdominal CT scan or MRI.^23^ The image examinations were carried out every 6–8 weeks during ICIs treatment.^22^ The study complied with Taiwan NHI regulation; HBV viral load monitoring was done every 6 months while on NUCs treatment. For patients on ICI treatment without NUCs, HBV viral loads were monitored every 2–3 months in this study. Additional HBV DNA test was performed if a serum alanine aminotransferase (ALT) level exceeded 100 U/L with suspicion of HBV reactivation.^24 25^ According to this guidance, 45 had follow-up HBV DNA values.

### Definitions

HBV reactivation was defined as a 10-fold increase in HBV DNA from baseline, or reappearance of HBsAg in HBsAg-negative case, or HBV DNA from undetectable to higher than 1000 IU/mL.^11 26^ Hepatitis related to HBV reactivation was defined as a threefold or greater increase in serum ALT that exceeds 100 U/L.^24 25^ The upper normal limit of ALT was 40 U/L. The ALT flare was defined as a rise in ALT>5 fold upper limit of normal (ULN), which is classified as grade III hepatic toxicity by CTCAE 5.0 grading system. The ALBI score was calculated using the formula: (log10 bilirubin x 0.66) + (albumin x 0.085), where bilirubin is in umol/L and albumin in g/L; and the cut points of the ALBI grade were based on previous report.^27^

### Virological and biochemical tests

Serum HBV DNA level was measured by quantitative PCR method (Roche COBAS 6800 HBV test) with the lower detection limit of 10 IU/mL. Serum biochemistry tests were measured by systemic multiauto-analyzer (Technicon SMAC, Technicon Instruments, Tarrytown, New York, USA). Serum AFP level was measured by chemiluminescent microparticle immunoassay (Elecsys AFP Assay, Roche Diagnostics, Mannheim, Germany) with clinically reportable range from 0.908 to 1 998 000 ng/mL.

### Statistical analysis

Continuous variables were expressed as median (range), while categorical variables were analyzed as frequency and percentages. The Pearson X^2^ analysis or Fisher’s exact test was used to compare categorical variables, while the Student t-test or Mann-Whitney U test was applied for continuous variables. For all analyzes, p<0.05 was considered statistically significant. All statistical analyzes were performed using the Statistical Package for Social Sciences (SPSS V.17.0 for Windows, SPSS).

## Results

### Demographic characteristics of the study cohort

The patients were classified into three categories, including HBV DNA ≤100 IU/mL on NUCs therapy, HBV DNA >100 IU/mL on NUCs therapy and HBV without NUCs therapy. For the six patients without NUCs treatment, four had HBV viral load less than 2000 IU/mL; the other two had elevated HBV viral loads, but their ALT levels were less than 2 folds of ULN; which were not fulfilled the regulation of Taiwan NHI for NUCs treatment. No significant differences of liver reserves could be identified between these three groups. The patients with HBV DNA ≤100 IU/mL on antiviral therapy were significantly older (median age: 63.4 vs 58.0 and 56.5 years old, p=0.019) compared with the others. Besides, these patients had smaller tumor size (4.0 vs 8.7 and 8.8 cm, p<0.001), lower serum level of AFP (373.2 vs 1992.6 and 3082.2 ng/mL, p=0.028); and more of them had experienced surgical resection for HCC before ICI treatment (62.9% vs 21.1% and 50.0%, p=0.013). Generally, most of the patients (80%) were at BCLC stage C. The majority (73.3%) of them was within Child-Pugh class A; but 65.0% was classified beyond ALBI grade 1. Two patients presented with positive anti-HCV antibody. Both their HCV viral loads were undetectable throughout the ICI treatment. The median cycles and duration of ICI treatment were five cycles (1–35) and 2.1 months (0.5–24.5) for patients with HBV DNA ≤100 IU/mL on NUCs; five cycles (1 – 23) and 2.3 months (0.2–15.0) for HBV DNA >100 IU/mL on NUCs; nine cycles (4 – 19) and 5.1 months (1.6–13.6) for patients not on NUCs, respectively. In addition, the follow-up period of the three patient groups was 5.4 months (0.5–25.7), 3.9 months (0.2–17.9) and 10.4 months (4.0–24.8), respectively. The detailed baseline characteristics were presented in table 1.

**Table 1:** Characteristics of 60 HBV-HCC patients treated with ICIs

Characteristics	HBV DNA ≤100 IU/mL on NUCs	HBV DNA >100 IU/mL on NUCs	Patient with HBV without NUCs	P value
	(n=35)	(n=19)	(n=6)	
Age, year	63.4 (45.6–78.8)	58.0 (40.8–77.7)	56.5 (40.1–66.5)	0.019
Sex (male), n (%)	31 (88.6)	15 (78.9)	4 (66.7)	0.341
NUCs, ETV/TDF/TAF, n (%)	24/8/3 (68.6/22.9/8.6)	15/1/3 (78.9/5.3/15.8)	–	–
Anti-HCV positive, n (%)	1 (2.9)	0 (0)	1 (16.7)	0.136
Max. tumor size, cm	4.0 (1.0–12.1)	8.7 (3.3–17.0)	8.8 (1.6–14.4)	<0.001
Tumor >50% liver volume, n (%)	6 (17.1)	10 (52.6)	4 (66.7)	0.006
Multiple tumors, n (%)	32 (91.4)	19 (100.0)	6 (100.0)	0.324
Extrahepatic metastasis, n (%)	19 (54.3)	8 (42.1)	2 (33.3)	0.514
Portal vein invasion, n (%)	16 (45.7)	4 (73.7)	5 (83.3)	0.058
AFP, ng/mL	373.2 (1.8–272 689.4)	1992.6 (13.7–785 992.2)	3082.2 (1076.2–1 148 415.7)	0.028
BCLC stage B/C, n (%)	8/27 (22.9/77.1)	4/15 (21.1/78.9)	0/6 (0/100.0)	0.429
Prothrombin time, INR	1.16 (0.90–1.47)	1.10 (1.00–3.12)	1.09 (0.97–1.42)	0.600
Platelet count, K/cumm	122 (43–360)	182 (71–553)	148 (128–367)	0.057
ALT, U/L	35 (11–254)	47 (17–213)	39 (22–64)	0.448
AST, U/L	42 (16–366)	90 (27–480)	61 (29–140)	0.106
Total bilirubin, mg/dL	0.69 (0.22–2.41)	1.10 (0.25–10.08)	0.97 (0.29–1.44)	0.854
Albumin, g/dL	3.8 (2.7–4.9)	3.6 (2.3–4.4)	3.5 (3.2–4.0)	0.106
Neutrophil, /cumm	4800 (2300–11900)	5550 (2500–12300)	5500 (3900–11600)	0.258
Neutrophil-lymphocyte ratio	3.89 (1.46–11.01)	3.90 (2.52–15.13)	4.45 (2.03–10.68)	0.684
Presence of ascites, n (%)	11 (31.4)	9 (47.4)	4 (66.7)	0.194
Child-Pugh score	6 (5–9)	6 (5–12)	6 (5–7)	0.368
Child-Pugh class A/B/C, n (%)	28/7/0 (80.0/20.0/0)	12/6/1 (63.2/31.6/5.3)	4/2/0 (66.7/33.3/0)	0.475
ALBI grade 1/2/3, n (%)	15/18/2 (42.9/51.4/5.7)	4/12/3 (21.1/63.2/15.8)	2/4/0 (33.3/66.7/14.3)	0.386
ICI treatment cycle	5 (1–35)	5 (1–23)	9 (4–19)	0.232
ICI treatment duration, months	2.1 (0.5–24.5)	2.3 (0.2–15.0)	5.1 (1.6–13.6)	0.257
Prior therapy to ICI, n (%)				
Surgical resection	22 (62.9)	4 (21.1)	3 (50.0)	0.013
RFA	16 (45.7)	3 (15.8)	2 (33.3)	0.088
TACE	22 (62.9)	8 (42.1)	5 (83.3)	0.143
Sorafenib	19 (54.3)	10 (52.6)	5 (83.3)	0.378
Combined ICI with TKI, n (%)	9 (25.7)	2 (10.5)	2 (33.3)	0.605
Immune-related AEs, n (%)	6 (17.2)	3 (15.8)	0 (0)	0.550
Follow-up period, months	5.4 (0.5–25.7)	3.9 (0.2–17.9)	10.4 (4.0–24.8)	0.199

Continuous variables are expressed as median (range).

AEs, adverse events; AFP, alpha-fetoprotein; AL(S)T, alanine (aspartate) aminotransferase; ETV, entecavir; ALBI grade, albumin-bilirubin grade; HBV, hepatitis B virus; HCV, hepatitis C virus; ICI, immune checkpoint inhibitor; INR, international normalized ratio; NUCs, nucleos(t)ide analogs; RFA, radiofrequency ablation; BCLC stage, Barcelona Clinic Liver Cancer stage; TACE, transarterial chemoembolization; TAF, tenofovir alafenamide; TDF, tenofovir disoproxil fumarate; TKI, tyrosine kinase inhibitor.

### Viral kinetics of HBV during ICI treatment

**Figure 1:**
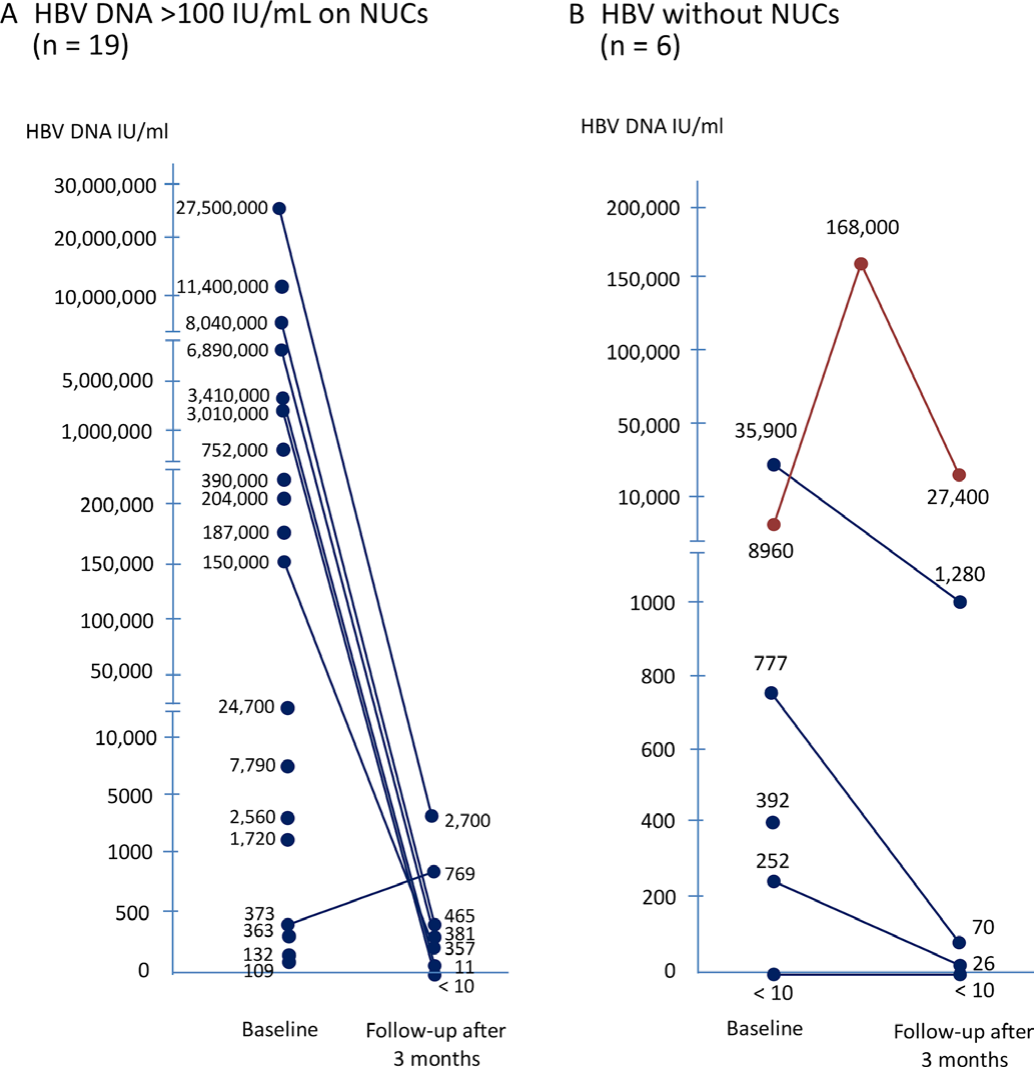
Kinetics of HBV DNA during ICI treatment. Kinetics of HBV DNA during ICI treatment in (A) patients with HBV DNA >100 IU/mL on NUCs, and (B) patients with HBV not on NUCs. Of 19 patients with HBV DNA >100 IU/mL on antiviral therapy, nine developed early tumor progression and short survival (<3 months), and three did not have significant ALT elevation during ICI treatment so that their followed data of HBV viral load were unavailable. ALT, alanine aminotransferase; HBV, hepatitis B virus; ICI, immune checkpoint inhibitor; NUCs, nucleos(t)ide analogs.

**Figure 2:**
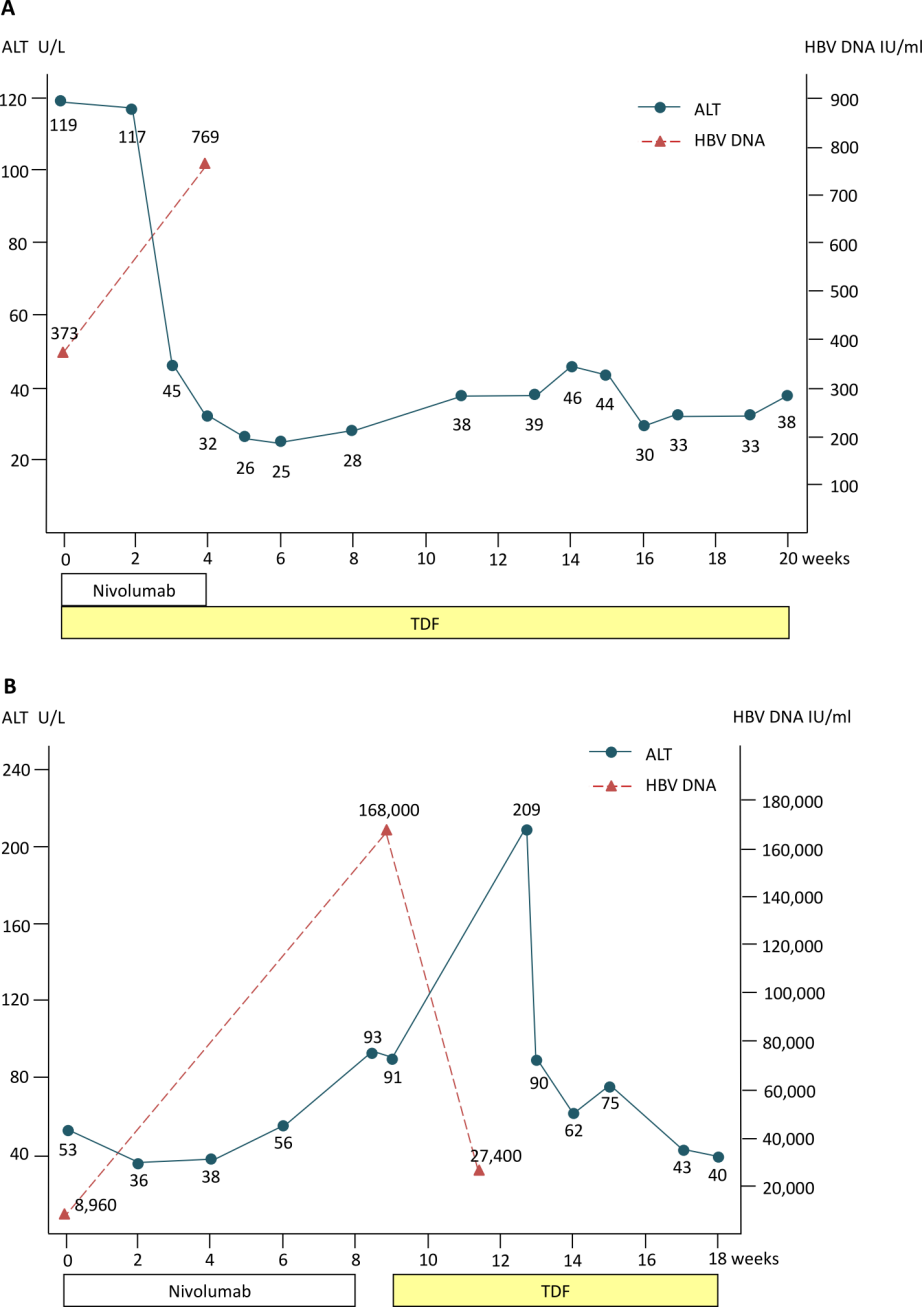
Kinetics of serum alanine aminotransferase (ALT) of two patients with hepatitis B virus (HBV) DNA elevation Kinetics of serum ALT of patients with HBV DNA elevation during ICI treatment who was classified in patients with HBV DNA >100 IU/mL on NUCs (A), and patients with HBV not on NUCs (B), respectively. ICI, immune checkpoint inhibitor; NUCs, nucleos(t)ide analogs; TDF, tenofovir disoproxil fumarate.

In patients with HBV DNA ≤100 IU/mL on NUCs therapy, no case experienced HBV reactivation during the ICIs treatment. Of the 19 patients with HBV DNA >100 IU/mL who started antiviral therapy simultaneously with ICI treatment, seven cases had longer survival to monitor their HBV DNA after NUCs treatment (figure 1A). Only one experienced suboptimal suppression by tenofovir with low HBV viremia but without ALT elevation or adverse events (details were presented in figure 2A); the others had a substantial decrease in HBV viral load during the follow-up period (table 2).

**Table 2:** HBV status during immune checkpoint inhibitors therapy

N (%)	HBV DNA ≤100 IU/mL on NUCs (n=35)	HBV DNA >100 IU/mL on NUCs (n=19)	Patients with HBV without NUCs (n=6)
Baseline HBV DNA			
Undetectable	31 (88.6)	0	1 (16.7)
Median (range) for detectable cases, IU/mL	41 (12–82)	187 000 (109 –27 500 000)	777 (252–35 900)
**HBV reactivation**	0	0	1
HBV DNA during ICI treatment			
≥1 log_10_ elevation	0	0	1 (16.7)
≥2 log_10_ elevation	0	0	0
Undetectable to detectable	3 (8.6)	0	0
Undetectable to >1000 IU/mL	0	0	0
**Peak HBV DNA** during ICI, IU/mL. median (range)	<10 (<10–1130)	381 (<10–2700)	70 (<10–1 68 000)
Hepatitis flare			
ALT >100 U/L	10 (28.6)	11 (57.9)	2 (33.3)
ALT >5X ULN	5 (14.3)	4 (21.1)	1 (16.7)
ALT >10X ULN	2 (5.7)	2 (10.5)	0 (0)
Icteric flare*	5 (14.3)	6 (31.6)	2 (33.3)
HBV DNA elevation and ALT >100 U/L	0	0	1 (16.7)
iRAE hepatitis	1 (2.9)	1 (5.3)	0 (0)

*Icteric flare is defined as serum ALT raised >3X ULN together with serum total bilirubin >2X ULN.

ALT, alanine aminotransferase; HBV, hepatitis B virus; ICI, immune checkpoint inhibitor; iRAE, immunotherapy related adverse event; NUCs, nucleos(t)ide analogs; ULN, upper limit of normal.

Of the six patients without antiviral treatment, five had detectable HBV viral load at baseline. Interestingly, three patients developed a notable decrease in HBV viral load during ICI treatment; and one maintained his serum HBV DNA in undetectable level during ICI treatment. Eventually, one patient experienced HBV reactivation with a >10 fold increase in HBV DNA level and ALT flare after 9 weeks of ICI treatment (figure 1B). After HBV reactivation, his HBV was controlled by tenofovir treatment and did not lead to HBV-related liver failure (table 3 and figure 2B). On the other hand, case N6 died of rapid tumor progression, and we could not get the followed data of HBV viral load.

**Table 3:** Clinical characteristics of the six patients without pre-emptive nucleot(s)ide treatment

Patient no	Age (years)	Sex	Baseline HBsAg (IU/mL)	Baseline HBV DNA (IU/mL)	Baseline AFP (ng/mL)	ICI ±TKI therapy	FU HBsAg (IU/mL)	FU HBV DNA (IU/mL)	FU AFP at BR (ng/mL)	Peak ALT (U/L)	Peak t.bili (mg/dL)	Rescue NUCs	Death caused by HBV	Best Tumor response to ICI
N1	40.9	M	26.4	35 900	2719.2	Nivolumab	2.01	1280	2799.9	85	0.63	–	None	PD
N2	66.5	F	27.8	252	1859.3	Nivolumab	58.2	26	4974.5	202	5.23	–	None	PD
N3	49.2	M	559.8	8960	1148415.8	Nivolumab	Missing	168 000	544 176.1	174	19.75	TDF	None	PD
N4	40.1	F	1856.77	777	1076.2	Nivolumab Lenvatinib	Missing	70	179.2	46	0.52	–	None	CR
N5	63.9	M	557.88	<20	3445.2	Nivolumab Regorafenib	Missing	<20	8905.3	37	2.77	–	None	PD
N6	64.1	M	0	392	129 258.1	Nivolumab	Missing	Missing	76 472.3	26	9.49	–	None	PD

AFP, alpha-fetoprotein; ALT, alanine aminotransferase; BR, best response; F, female; HBsAg, HBV surface antigen; HBV, hepatitis B virus; ICI, immune checkpoint inhibitor; M, male; NUCs, nucleos(t)ide analogs; T.bili, total bilirubin; TDF, tenofovir disoproxil fumarate; TKI, tyrosine kinase inhibitor.

### Status of hepatitis during ICI treatment

Twenty-three patients (38.3%) experienced ALT>100 U/L during the follow-up period. Of them, 10 patients had ALT flare (>5 fold ULN), but only four had more than 10-fold ULN ALT increase (5.7% and 10.5% in HBV DNA ≤100 IU/mL and HBV DNA >100 IU/mL on NUCs patients) (table 2). As presented in table 4, tumor progression is the main cause of ALT elevation in each group of patients. Two patients on NUCs therapy (one with baseline HBV DNA ≤100 IU/mL and the other one>100 IU/mL) had developed immune-related adverse event (iRAE) hepatitis. The incidence of iRAE hepatitis was not significantly different among the three groups. Of the six HBV patients without NUCs treatment, two developed ALT elevation >100 U/L during ICI therapy. One was resulted from HBV reactivation, and the other one was attributed to tumor progression.

**Table 4:** Causes of ALT >100 U/L in HBV-HCC patients on ICI treatment

N (%)	HBV DNA ≤100 IU/mL on NUCs (n=10)	HBV DNA >100 IU/mL on NUCs (n=11)	Patients with HBV without NUCs (n=2)
Tumor progression	9 (90.0)	10 (90.9)	1 (50.0)
HBV reactivation	0	0	1 (50.0)
iRAE hepatitis	1 (10.0)	1 (9.1)	0 (0)

ALT, alanine aminotransferase; HBV, hepatitis B virus; ICI, immune checkpoint inhibitor; iRAE, immunotherapy related adverse event; NUCs, nucleos(t)ide analogs.

## Discussion

This is the largest real-world cohort from Asian patients with HBV-related HCC treated by ICIs till now. In our cohort, HBV reactivation developed in only one patient who did not receive antiviral agent during ICI therapy; and was controlled by tenofovir treatment that did not lead to HBV-related liver failure. Otherwise, no patients with ongoing NUCs suffered from noteworthy HBV reactivation during ICI treatment; even their baseline HBV DNA levels were higher than 100 IU/mL.

For patients with HBV-related HCC, HBV reactivation and its subsequent morbidity were identified as poor prognostic factors of the overall survival.^28^ According to previous studies, the incidence of HBV reactivation ranges from 4% to 67% in patients with HBV-related HCC undergoing chemotherapy; and the contributed mortality rate was up to 18%.^29 30^ Apart from the direct cause of death, the scheduled treatment to HCC would be delayed or premature terminated due to deteriorated liver function by HBV reactivation that would also have impacts on the prognosis.^31 32^ For patients who received hepatectomy or loco-regional therapies for HCC, HBV reactivation was also reported and identified in relation to the prognosis.^33–35^ However, the data regarding to the kinetics of HBV viral loads during ICI therapy for HCC were still limited.

In patients with chronic hepatitis B, the upregulated inhibitory receptors on the CD8 T cells limit their defensive function and lead to the exhausted phenotype.^13^ PD-1 is the most expressed inhibitory receptor, especially on the intrahepatic HBV-speciﬁc T cells.^36 37^ Blockage of PD-1/PD-L1 pathway could restore not only antitumor but also antiviral T cell function then help to suppress HBV viral load.^12 37^ According to the recent phase 1b study, a single dose of nivolumab at 0.3 mg/kg could lead to HBsAg decline in HBeAg-negative chronic hepatitis B.^38^ Despite the lack of data to support HBsAg decline in our study, we still demonstrated three out of six cases had substantial decline in HBV DNA and additional one case maintained his HBV viral load in undetectable status during ICI therapy among patients who did not receive NUCs treatment. These findings may imply the potential role of ICI in the treatment of chronic hepatitis B. However, even under NUCs treatment, a more than 1 log increase in HBV DNA during nivolumab treatment was reported in 11% (5/47) of HBV-related HCC patients in the Asian cohort of checkmate 040.^39^ In spite of that, their peak HBV viral load were all less than 2000 IU/mL and no HBV-related adverse events were reported. In our cohort, presence of low HBV viremia was observed in one patient during tenofovir treatment which did not lead to ALT elevation or adverse events. According to AASLD (American Association for the Study of Liver Diseases) guideline, change NUCs is not required as it did not meet the definition of antiviral drug resistance (1 log increase in HBV DNA).^11^ In addition to the previous case reports,^17 18 40^ a recent Chinese study demonstrated that 6 of 114 (5.3%) HBsAg-positive patients with various cancers developed HBV reactivation during anti-PD-1/PD-L1 treatment, but no HBV-related fatal events occurred.^41^ Our real-world data also demonstrated that HBV reactivation would occur in one out of six patients who did not receive pre-emptive anti-viral treatment. Accordingly, we still have to keep awareness of HBV reactivation during ICI treatment for HBV-HCC.^42^

In checkmate-040 and Keynote-224 trials, patients were required to receive effective anti‐viral therapy and had a viral load <100 IU/mL before initiating ICI treatment.^5 6^ In the real life, however, ICIs would be delivered to more complex populations of patients than those of clinical trials that raise a question to the necessity of keeping low viral load before ICI therapy. Our data suggested that HBV viral load higher than 100 IU/mL is safe and not a contraindication for ICI treatment for HCC, if NUCs can be coadministrated. By this way, none developed HBV viral load increase during the treatment course.

Although our data supported the feasibility of administering anti-PD-1 therapy in patients with HCC and HBV, the optimal use of NUCs remains uncertain. Whether patients at low risk of HBV reactivation may be monitored rather than receive prophylaxis in this setting, and an optimal threshold of HBV viral load that ICIs can be safely initiated without NUCs required further studies to determine.

There are several limitations in this study. First, this study only enrolled patients from a single hospital. However, our hospital is the main leading tertiary medical center in Taiwan. The information bias would be ameliorated by regular clinical assessment. Besides, it is so far the largest real-world HBV-HCC cohort that investigated the viral kinetics of HBV during ICI treatment. Second, the serum level of HBsAg was not frequently rechecked in our patients during ICI treatment according to the reimbursement criteria of NHI in Taiwan. Third, no ideal control arm could be designed in this study. For sorafenib-failed HCC patients, sorafenib on treatment group could not serve as control. For patients who received ICIs as first-line therapy, sorafenib on treatment patients were also not suitable as a control group because the pharmacologic difference between sorafenib and ICIs. The ideal control group should be HCC patients with serum HBV DNA >100 IU/mL who received ICI treatment after sorafenib failure or intolerance but was prohibited to use NUCs throughout the treatment. However, it is not ethical for this design. Forth, the risk of HBV reactivation might be underestimated because some patients only received very few cycles of ICIs and experienced early tumor progression and mortality. In our study, 15 did not have follow-up HBV DNA values; including nine patients did not experience ALT elevation during ICIs treatment; and the other six patients met early tumor progression. The risk of HBV reactivation might be underestimated in these cases. Finally, the antiviral agents prescribed to our patients were not consistent. Most of the patients (72.2%) on antiviral therapy were prescribed with entecavir; and the others used tenofovir disoproxil fumarate or tenofovir alafenamide. However, all these NUCs belong to high potency antiviral agents that could suppress high HBV viral loads.^43 44^

In conclusion, no patients on antiviral therapy (regardless of HBV viral load at baseline) developed HBV reactivation, and one out of six not receiving NUCs had HBV reactivation. Patients with HBV viral load higher than 100 IU/mL could safely receive ICI treatment under the protection of NUCs treatment.
